# Quantitative characterization of retinal features in translated OCTA

**DOI:** 10.3389/ebm.2024.10333

**Published:** 2024-10-23

**Authors:** Rashadul Hasan Badhon, Atalie Carina Thompson, Jennifer I. Lim, Theodore Leng, Minhaj Nur Alam

**Affiliations:** ^1^ Department of Electrical and Computer Engineering, University of North Carolina at Charlotte, Charlotte, NC, United States; ^2^ Department of Surgical Ophthalmology, Atrium-Health Wake Forest Baptist, Winston-Salem, NC, United States; ^3^ Department of Ophthalmology and Visual Science, University of Illinois at Chicago, Chicago, IL, United States; ^4^ Department of Ophthalmology, Stanford University School of Medicine, Stanford, CA, United States

**Keywords:** GAN, generative AI, OCT, OCTA, OCTA features

## Abstract

This study explores the feasibility of quantitative Optical Coherence Tomography Angiography (OCTA) features translated from OCT using generative machine learning (ML) for characterizing vascular changes in retina. A generative adversarial network framework was employed alongside a 2D vascular segmentation and a 2D OCTA image translation model, trained on the OCT-500 public dataset and validated with data from the University of Illinois at Chicago (UIC) retina clinic. Datasets are categorized by scanning range (Field of view) and disease status. Validation involved quality and quantitative metrics, comparing translated OCTA (TR-OCTA) with ground truth OCTAs (GT-OCTA) to assess the feasibility for objective disease diagnosis. In our study, TR-OCTAs showed high image quality in both 3 and 6 mm datasets (high-resolution and contrast quality, moderate structural similarity compared to GT-OCTAs). Vascular features like tortuosity and vessel perimeter index exhibits more consistent trends compared to density features which are affected by local vascular distortions. For the validation dataset (UIC), the metrics show similar trend with a slightly decreased performance since the model training was blind on UIC data, to evaluate inference performance. Overall, this study presents a promising solution to the limitations of OCTA adoption in clinical practice by using vascular features from TR-OCTA for disease detection. By making detailed vascular imaging more widely accessible and reducing reliance on expensive OCTA equipment, this research has the potential to significantly enhance the diagnostic process for retinal diseases.

## Impact statement

This study represents a significant advancement in retinal imaging by demonstrating the feasibility of using generative machine learning to translate OCT features into OCTA features, addressing a critical gap in clinical practice. By employing a generative adversarial network framework trained on diverse datasets, the research establishes quantitative features in Translated OCTA. This innovation enhances the ability to objectively diagnose retinal diseases by providing reliable vascular imaging without the need for costly OCTA equipment. The findings reveal that vascular features from TR-OCTA, such as tortuosity and vessel perimeter index, offer more consistent diagnostic trends compared to traditional density features. This new information has the potential to transform retinal disease diagnostics, making detailed vascular imaging more accessible and cost-effective, thereby improving patient outcomes and broadening the adoption of advanced imaging techniques in routine clinical settings.

## Introduction

Optical Coherence Tomography (OCT) is a cutting-edge medical imaging technology that has revolutionized our ability to observe and comprehend the complex structures of biological tissues. It is non-invasive and capable of providing highly detailed in-depth retinal pathologies. It generates high-resolution cross-sectional images of tissues using low-coherence light, therefore has been widely adopted in ophthalmic clinical care [1]. As a result, OCT has been demonstrated for early identification and monitoring of various retinal illnesses including diabetic retinopathy (DR), age-related macular degeneration (AMD) and glaucoma that cannot be obtained by any other non-invasive diagnostic technique [2–8].

The rapid development of OCT, growing interest in this field, and its increasing impact in clinical medicine has contributed to its widespread availability. However, due to its non-dynamic imaging technology, conventional OCT cannot visualize blood flow information such as blood vessel caliber or density and remains only limited to capturing structural information [2, 9]. As a result of this information gap, OCT angiography (OCTA) was developed which can produce volumetric data from choroidal and retinal layers and provide both structural and blood flow information [10, 11]. OCTA provides a high-resolution image of the retinal vasculature at the capillary level, allowing for reliable detection of microvascular anomalies in diabetic eyes and vascular occlusions. It helps to quantify vascular impairment based on the severity of retinal diseases. In recent years, OCTA has been demonstrated to identify, detect, and predict DR [12–19], AMD [20–22], Glaucoma [23] and several other retinal diseases [24–31]. Despite the advantages, widespread deployment of OCTA has been limited due to the high device cost [32, 33]. The additional requirements of hardware and software for an OCTA device pose a financial burden for clinics as well as patients This is one of the major reasons that only a limited number of hospitals and retinal clinics use OCTA for in-depth retinal vascular analysis. Another limitation of OCTA is the process of generating an OCTA scan, which takes longer time and involves repetitive scanning of the retina making the data acquisition harder due to involuntary eye movements and motion artifacts, reducing the quality of OCTA images [33]. Due to the limitation of OCTA data, most studies involving OCTA based imaging biomarkers and involving the use of artificial intelligence (AI) are difficult to validate extensively for future clinical deployment.

From literature, a potential solution to this problem can be the utilization of AI and machine learning (ML) to produce OCTA images from the already available OCT data which has been showing promising outcomes [34–39]. Incorporating ML for OCTA translation from OCT offers significant advances in ophthalmic diagnostics by increasing angiographic and functional information in existing OCT data. This transition harnesses ML’s capability to autonomously analyse OCT scans and generate detailed vascular images, traditionally obtained through OCTA, aligned with OCT information. By doing so, it substantially lowers the barriers to accessing high-resolution vascular imaging, which is crucial for diagnosing and monitoring retinal diseases and provides a robust detection system. Furthermore, ML dependent approaches alleviate some of OCTA’s limitations, including its high cost, susceptibility to artifacts from patient movement and the extensive time required for image acquisition.

Different studies have been reported [40–42] attempting to leverage ML algorithms for generative-adversarial learning, typically utilizing a UNet for image translation in recent years. However the quality of the translated OCTA (TR-OCTA) is usually sub-optimal and the retinal vascular areas are not refined enough. The first application of this approach was reported by Lee et al., 2019 [34] to train an algorithm to generate retinal flow maps from OCT images avoiding the needs for labelling but it was limited to capture higher density of deep capillary networks. According to some recent studies [35–37], incorporating textual information or surrounding pixels, it is possible to improve the OCTA image quality. Le et. Al [39] proposed another approach incorporating spatial speckle variance and generative AI, however, it requires OCT/OCTA data from custom devices. In this paper, we adopt and implement a generative-adversarial learning framework-based algorithm demonstrated by Li et al [36] for translating OCT data into OCTA. The focus of this study is to demonstrate the feasibility of using such TR-OCTA image generated vascular features (Blood Vessel Density (BVD), Blood Vessel Caliber (BVC), Blood Vessel Tortuosity (BVT), Vessel Perimeter Index (VPI)) for disease detection. We compare these OCTA features with ground truth (GT) – OCTAs. The quality of the TR-OCTAs were compared with features such as Structural Similarity Index Measure (SSIM), Fréchet Inception Distance (FID) and patch-based contrast quality index (PCQI). From our observation and statistical analysis, we found that overall, the SSIM values indicate a moderate level of structural similarity between TR-OCTA and GT-OCTA images, with some variability across different patient categories and scan range however PCQI scores are quite close for both dataset and some deviation in FID scores is noticeable. It was observed that the model generally achieved a slightly better performance in depicting normal and pathological retinal features for the 3 mm scans compared to the 6 mm. However, across both field of view (FoV), there were slight discrepancies in quantitative vascular metrics such as BVD, BVC and VPI, highlighting areas where the translation model could be further refined. This analysis underscores the potential of using AI-driven translation models for OCTA image analysis, while also pointing to the need for improvements to enhance the accuracy of vascular feature representation, particularly at varying FoV.

## Materials and methods

**FIGURE 1:**
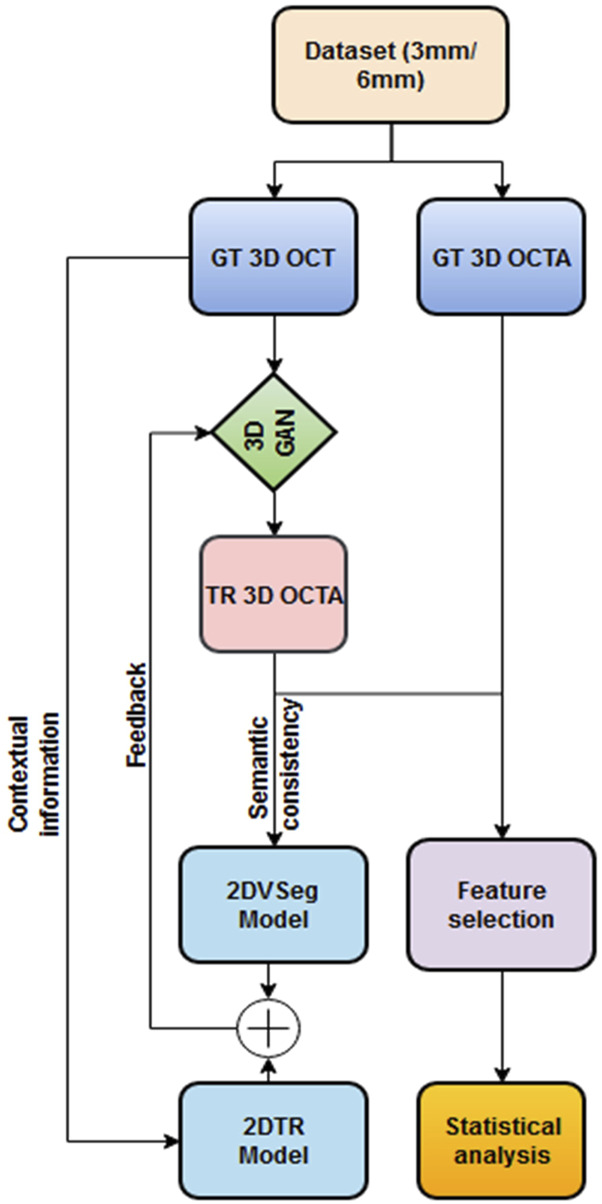
Framework of OCT to OCTA translation and characterization of quantitative features.

The overall methodology of our feature extraction pipeline is demonstrated in Figure 1. We first translate OCT data into OCTA (using algorithm demonstrated by Li et. al [36]) and quantify the retinal features in both GT and TR-OCTAs for validation.

### Translation algorithm

**FIGURE 2:**
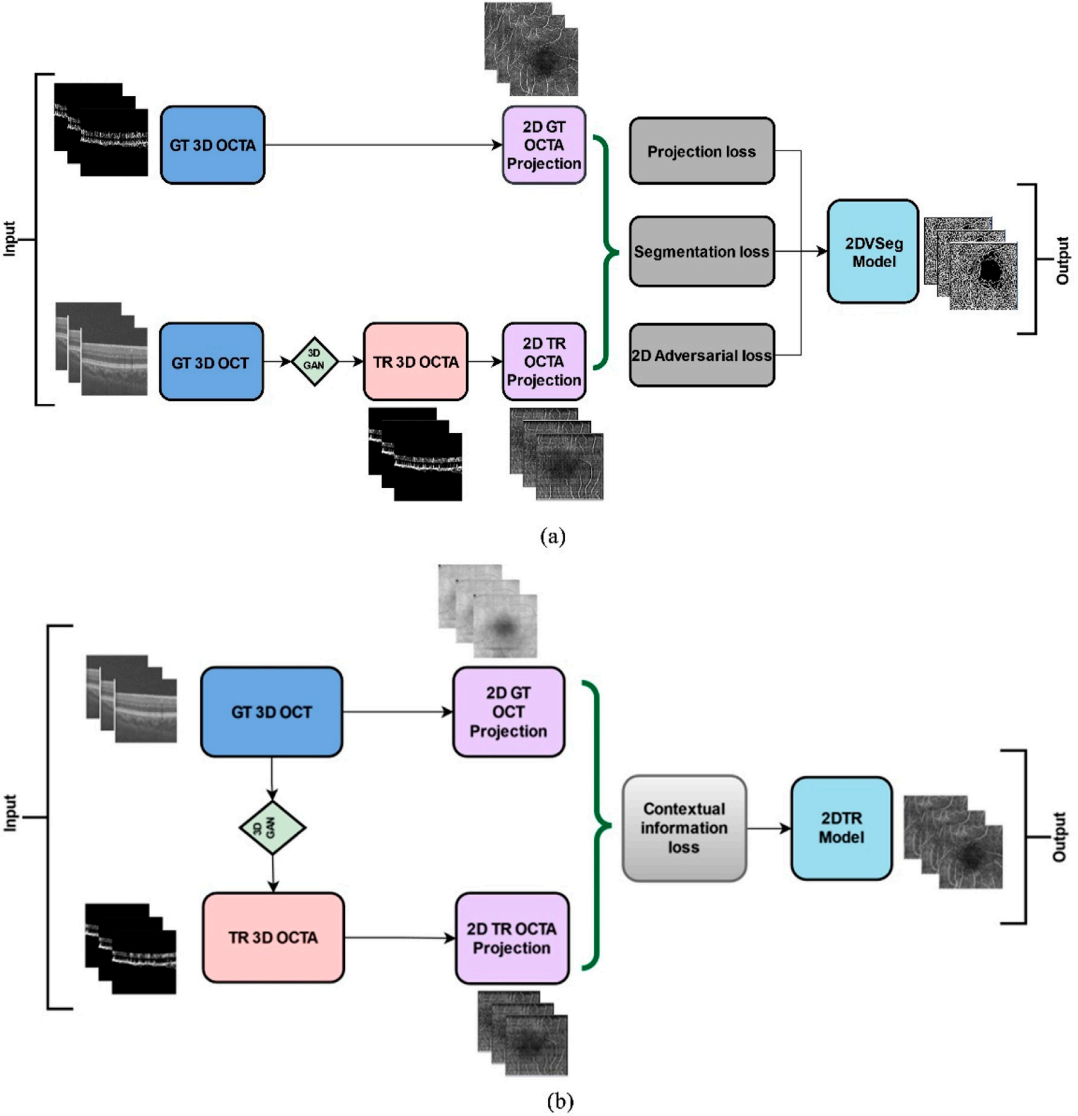
**(A)** 2D vascular segmentation model, **(B)** 2D Translation model.

We adopted and implemented the OCT to OCTA translation algorithm from Li et al [36]. We describe the process here briefly. The process of OCTA translation from OCT images is carried out in 3 steps (Figure 1): (a) generating 3D OCTA volumes from paired 3D OCT volumes using conditional generative adversarial network (GAN), (b) improving image quality by focusing only the vascular regions, utilizing the 2DVSeg model, thorough vascular segmentation, (c) preserving contextual information for better quality translated images through a 2D translation model (2DTR) generating 2D paired OCTA maps. The baseline architecture of the translation model is built upon pix2pix, an image translation model [41]. The aim of the model is primarily to translate OCT volumes to its paired OCTA volume as closely as possible to the original clinical images**.** [4] The framework includes a 3D GAN where the 3D generator takes a 3D OCT volume as its input and outputs a corresponding TR-OCTA volume. a 3D discriminator is used to effectively distinguish between the original (ground-truth) OCTA volumes and the generated ones. An adversarial loss is used to train both the generator and discriminator. Furthermore, to calculate for each pixel difference between TR-OCTA and GT-OCTA, a distance loss is considered. The framework also uses a 2D vascular segmentation model (Figure 2A) to help with the improved quality of the vascular regions by utilizing OCTA reflected vascular data by focusing on the vascular areas during the 3D volume translation process.

This model also utilizes a 2D generative translation model (Figure 2B) to build heuristic (suboptimal) 2D OCTA projection maps from their corresponding OCT that can provide heuristic contextual information where output values are affected by the surrounding pixels resulting in outputs with additional contextual information.

### Comparative feature analysis

The generated TR-OCTA maps were compared on several quantitative features to the GT projection maps for comparison: BVD, BVC, BVT, and VPI. Also, for qualitative comparison: SSIM, FID and PCQI metrics were used to quantify the translated image quality and similarity to GT OCTA maps. All the metrics evaluation were performed using MATLAB and Python. Feature values were calculated separately for 3 mm and 6 mm across different patient groups and compared between the OCT500 and UIC datasets. A two-tail t-test was carried out for each feature to compare if there is a significant difference between the TR-OCTA and GT-OCTA values with a p value <0.05.

### Metrics and features

#### Similarity metrics

We used three metrics to compare GT and TR-OCTAs, as described below:

SSIM: SSIM or Structural Similarity Index Measure, is a method for measuring the similarity between two images. SSIM is based on the perception of the human visual system and it considers changes in structural information, luminance and contrast. The idea is that pixels have strong inter-dependencies, especially when they are spatially close. These dependencies carry important information about the structure of the objects in the visual scene.

FID: FID score is a metric used to evaluate the quality of images generated by models, such as those produced by GANs. It measures the similarity between two sets of images, typically between a set of generated images and a set of real images, by comparing the statistics of their features extracted by a pre-trained Inception model [43]. The FID score calculates the distance between the feature vectors of the real and generated images, using the Fréchet distance (also known as the Wasserstein-2 distance). A lower FID score indicates that the distribution of the generated images is closer to the distribution of the real images, suggesting higher quality and more realistic images.

PCQI: PCQI is another metric designed to assess the quality of images by focusing on local contrast changes, which are crucial for visual perception, especially in textured regions [44]. Unlike many traditional image quality metrics that evaluate images globally, PCQI operates on small, localized patches of an image, making it particularly effective at capturing and evaluating detailed contrast differences between a reference image and a test image. PCQI calculates the quality score based on three main aspects: patch similarity, contrast distortion, and mean luminance change, within these localized regions. The final score is a weighted sum of these aspects, providing a single quality metric that reflects how perceptually close the test image is to the reference image in terms of local contrast and brightness. A higher PCQI score indicates a better match between the test and reference images, suggesting less contrast distortion and more accurate reproduction of the original image’s visual quality.

#### Quantitative OCTA features

We characterized three vessel and one density based features (Equations 1–4), as described below:

BVD: BVD or vessel area density (VD) [45], is the ratio of the blood vessels to the total area measured and can be utilized for identifying early detection of retinal pathologies including DR [46, 47], AMD [48, 49] etc.

$$BVD=\frac{vascular\;area}{total\;area}$$ (1)

BVC: BVC, also named as vessel diameter index [50], is calculated as the ratio of vessel area to the vessel length [12]. BVC distortion can be used to quantify retinal vascular shrinkage and is typically observed in different retinopathies such as diabetic retinopathy (DR) [51].

$$BVC=\frac{vascular\;area}{vascular\;length}$$ (2)

BVT: BVT is defined as a measure of degree of vessel distortion [26, 52]. During any retinal pathologies, distorted vessel structures can affect the blood flow efficiency and can be measured as:

$$BVT=\frac{1}{n}\sum \frac{geodesic\;distance\;between\;endpoints\;for\;a\;vessel\;branch}{euclidean\;distance\;between\;endpoints\;for\;a\;vessel\;branch}$$ (3)

here, n = total number of vessel branches.

VPI: VPI [52] is measured as the ratio of the contour length of the vessel boundaries or vessel perimeter to the total vessel area and has been used for detection of DR and sickle cell retinopathy (SCR) from.

OCTA images:

$$VPI=\frac{overall\;contour\;length\;of\;blood\;vessel\;boundaries}{total\;blood\;vessel\;area}$$ (4)

Statistical Analysis: We performed statistical analysis based on the selected features to quantify the TR-OCTA and measure the quality of the translation. This analysis will help us improve the accuracy and efficiency of the TR-OCTA translated from GT-OCT and GT-OCTA.

## Results

### Dataset

We used 2 datasets for our study, a public dataset of 500 patients containing paired 3D OCT and OCTA volumes, OCTA-500 [53] and a dataset of DR patients collected from UIC with 445 scans containing OCT volumes and OCTA projections.

OCT500 dataset is divided into 2 subsets according to their FoV (Field of view), 3 mm and 6 mm. The translation algorithm is applied separately to the two subsets for comparison. The datasets are further divided into different diseased patients and normal patients for quantitative feature comparison. The 3 mm dataset contains 6 AMD patients, 5 Choroidal neovascularization (CNV) patients, 29 DR and 160 Normal patients who are compared statistically after evaluating the feature values. The 3 mm set contains paired OCT and OCTA volumes from 200 patients with a field of view (FOV) 3 mm *×* 2 mm *×* 3 mm. Each volume has 304 slices with a size of 640px × 304px. The generated projection map is of 256px × 256px size. The whole dataset is divided into a 70-25-5% split: 140, 10, and 50 volumes for training, validation and test sets respectively. Similarly, the 6 mm set contains paired OCT and OCTA volumes from 300 patients with FOV of 6 mm *×* 2 mm *×* 6 mm. Each volume is of size 640px × 400px, containing 400 slices and generated projection maps are of size 256px × 256px. Similar to 3 mm set: 180, 20, and 100 volumes are split as training, validation and test sets. The 6 mm dataset contains 43 AMD, 11 CNV, 14 Central serous chorioretinopathy (CSC), 35 DR, 10 Retinal vein occlusion (RVO), 91 Normal and 96 other retinal pathology-affected patients for which a similar statistical evaluation is carried out and feature values are calculated.

#### UIC data and image acquisition

The UIC study was approved by the institutional review board of the University of Illinois at Chicago and was in compliance with the ethical standards stated in the Declaration of Helsinki. The patients with DR were recruited from UIC Retinal Clinic. We performed a retrospective study of consecutive diabetic patients (Type II) who underwent OCTA and OCT imaging. The patients are thus representative of a university population of diabetic patients who require imaging for management of diabetic macular edema and DR. OCT/OCTA images of both eyes of every patient were collected. We excluded subjects with macular edema, previous vitreous surgery, and history of other eye diseases. All patients had undergone a complete anterior and dilated posterior segment examination (J.I.L.). The patients were classified by severity of DR (mild, moderate, and severe) according to the Early Treatment Diabetic Retinopathy Study staging system. The grading was done by retina specialist who used a slit-lamp fundus lens for the diagnosis. OCT/OCTA data were acquired using an ANGIOVUE spectral domain OCTA system (Optovue, Fremont, CA), with a 70-kHz A-scan rate, an axial an axial resolution of 5 μm, and a lateral resolution of 15 µm. All the OCTA images were macular scans and had field of view of 6 mm. We exported the OCTA images from the software ReVue (Optovue) and used custom-developed software in Python OpenCV for further image analysis, feature extraction, and image classification.

The UIC dataset contains 445 OCT scans from 41 patients with different DR conditions: control, mild, moderate and severe. The scans were selected based on signal strength Q ≥ 5 for this study. Similar to OCT500, this dataset has both 3 mm (187 scans) and 6 mm (258 scans) scans for different stages of DR: Control, Mild, Moderate and Severe. For 3 mm FOV, we used 35 scans for Control group, 118 for Mild, 37 for Moderate and 97 for Severe. On the other hand, for 6 mm, the set included 59 for Control, 143 Mild, 69 Moderate and 123 Severe scans for comparison. 3 mm slices are of size 640px × 304px and 6 mm slices are mostly of 640px × 400px with some mixed 640px × 304px scans which are used to generate 256px × 256px OCTA slices. Some patients were listed in multiple categories therefore, scans of those patients were included in multiple categories before feature evaluation.

### Comparative analysis of similarity and OCTA features

**FIGURE 3:**
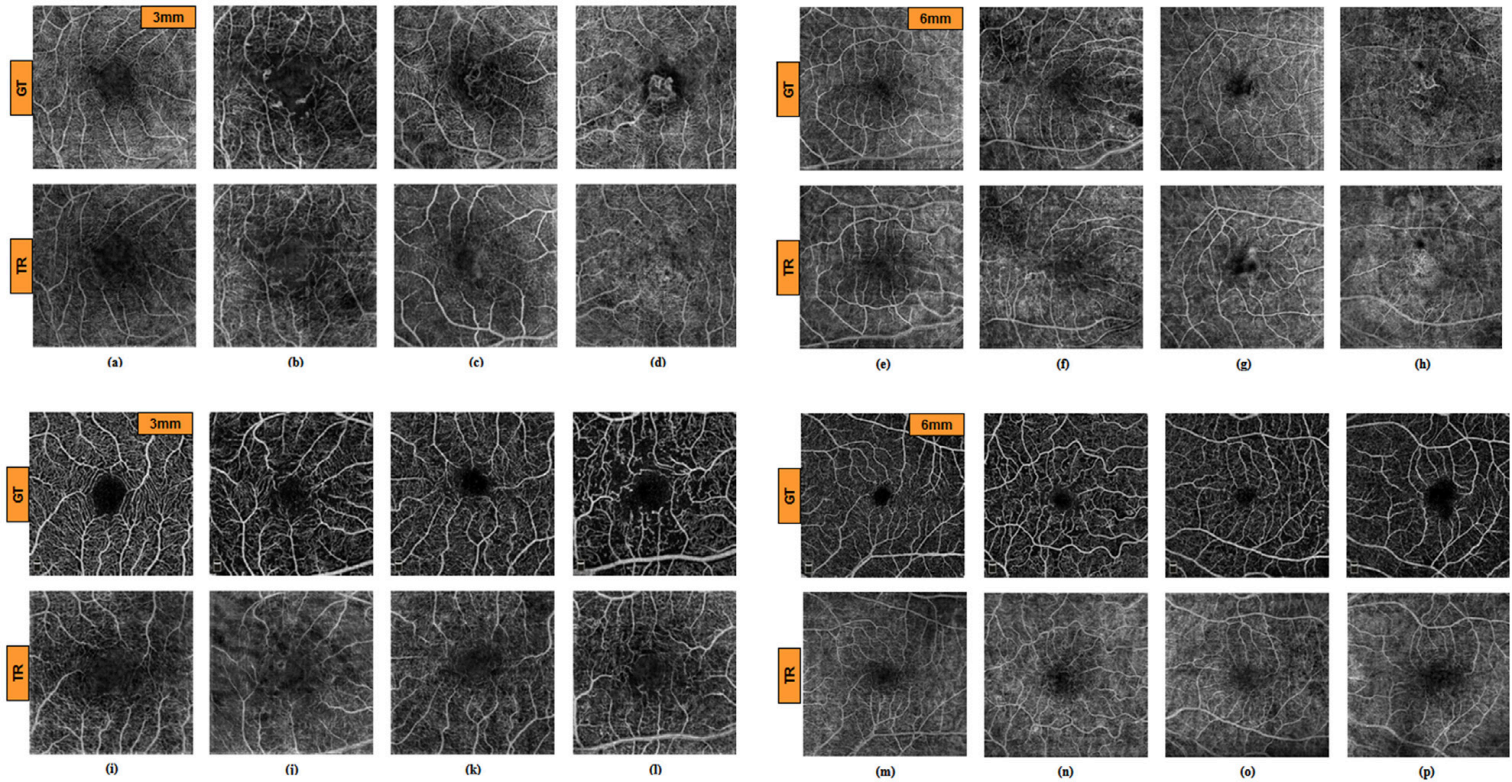
GT and TR-OCTA images for NORMAL **(A, E)**, DR **(B, F)**, CNV **(C, G)** and AMD **(D, H)** patients from OCT500. **(A–D)** images are of 3 mm and **(E–H)** are of 6 mm. Similarly **(I–P)** are OCTA images from UIC dataset. **(I, M)** from Control, **(J, N)** from Mild, **(K, O)** from Moderate and **(L, P)** from Severe group for 3 mm and 6 mm respectively.

The algorithm for TR-OCTA generation was exclusively trained only on OCT500 and tested on the UIC dataset for feature quantification and comparison. Figures 3A–H depicts GT-OCTA and generated TR-OCTA images at 3 mm and 6 mm scan range from diseased as well as normal patients for OCT500 while Figures 3I–P represent the images for UIC dataset across various patient categories.

**FIGURE 4:**
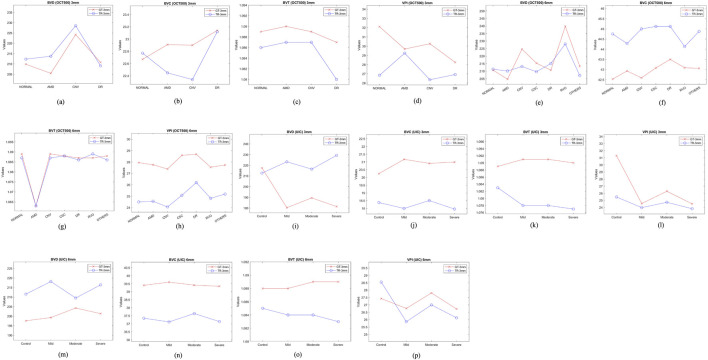
Feature values for different patient groups from both OCT500 and UIC. **(A–D)** show feature trend of 3 mm for OCT500, **(E–H)** for OCT500 6 mm, **(I–L)** UIC 3 mm and **(M–P)** UIC 6 mm dataset.

**FIGURE 5:**
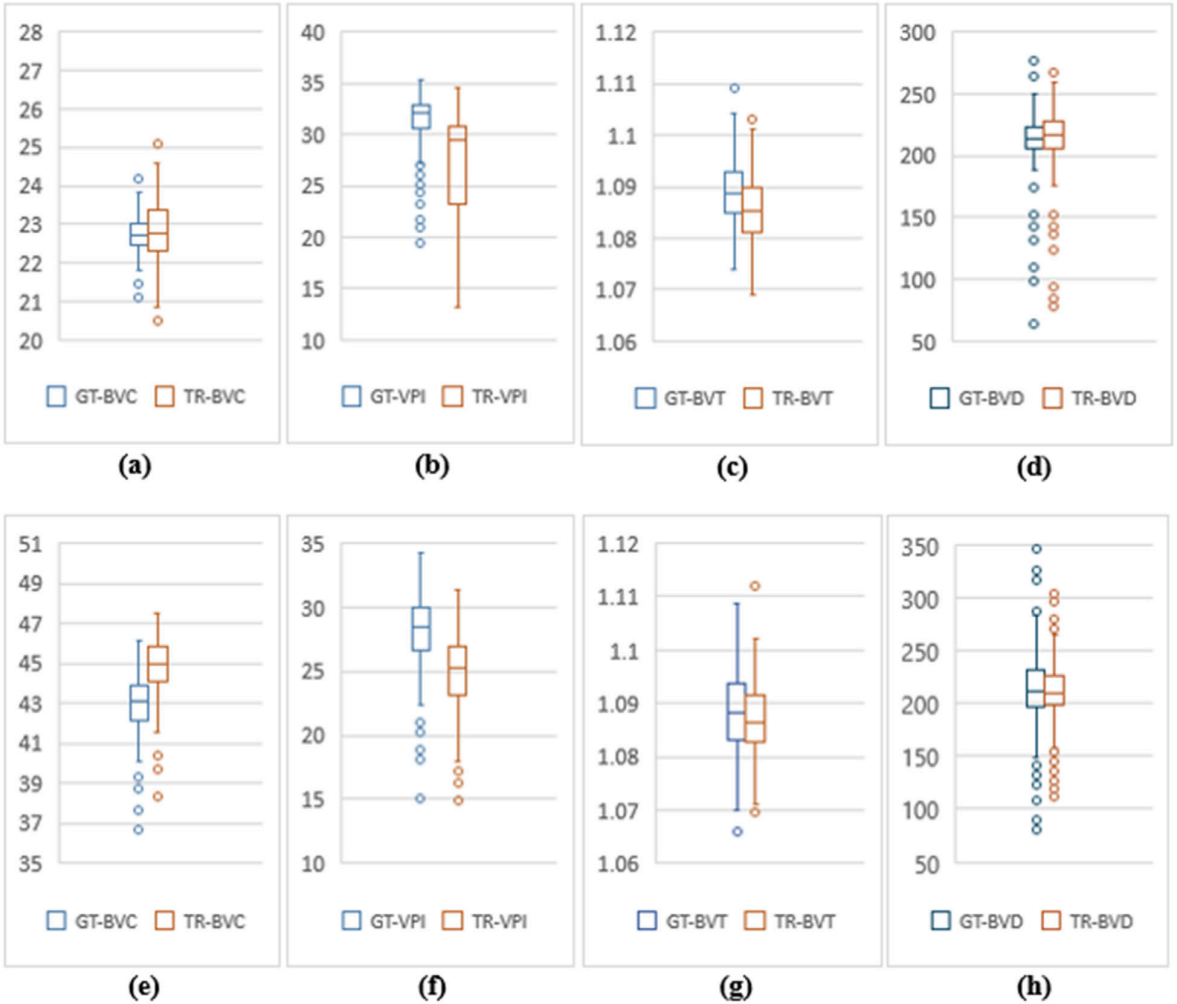
Feature value distribution of 3 mm and 6 mm scans for OCT500. **(A–D)** are BVC, VPI, BVT and BVD values for 3 mm. Similarly, **(E–H)** show values for 6 mm.

OCT500 3 mm dataset has AMD (6), CNV (5), DR (29) and NORMAL (160) patients totalling 200 patients, while 6 mm dataset includes AMD (43), CNV (11), CSC (14), DR (35), RVO (10), OTHER retinopathies (96) and NORMAL (91) patients totalling 300. Two-tail T-tests were carried out (p < 0.05) between GT and TR-OCTAs for BVD, BVC, BVT and VPI (3 mm complete dataset). The results indicate that BVD and BVC have p-values close to 0.5, suggesting that there is no significant difference between these features in the GT and TR-OCTA values (Table 1). This means that these features can be used for effective disease classification. Upon evaluating quality metrics for the datasets (Tables 2, 3), mean SSIM for 3 mm was found to be slightly higher (0.48) than 6 mm dataset showing SSIM ranging from 0.16–0.52 with a mean of 0.42. SSIM values were also calculated for different patient statuses in both datasets. For the 3 mm dataset: AMD patients show a slightly lower mean SSIM, DR dataset on the other hand reveals a higher mean SSIM compared to other patient groups. However, for 6 mm: AMD, CNV, CSC, patients with other retinopathies and Normal group showed a close SSIM mean value except for DR patients with a slightly higher mean SSIM (0.43) and RVO patients, a lower mean SSIM of 0.36 (Table 2). Furthermore, The FID score for the 3 mm dataset was lower (35.88) compared to the 6 mm dataset, which had a higher FID score of 49.06. On the other hand, PCQI scores were comparably high for both datasets with the 3 mm set slightly outperforming the 6 mm (Table 3). All these feature values were also calculated for the complete dataset and separately for different diseased and normal patients for comparative analysis (Table 4). BVD and BVT values from 3 mm show some trend among Normal and AMD, CNV, DR groups which is mimicked by the TR-OCTA (Figures 4A–D). Overall, TR-BVC, TR-VPI, TR-BVT, and TR-BVD values (Figure 5) are concentrated within a specific range and closer to the GT values for each feature respectively. For BVD, some outliers are further away from the lowest value of the BVD range which is consistent with the findings from the categorized distribution of feature values (Supplementary Figures S1A–D).

**TABLE 1:** Two-tail t-test between GT-OCTA and TR-OCTA for OCT500 and UIC datasets.

Quantitative Features	OCT500 3 mm (p< .05)	OCT500 6 mm (p < .05)	UIC 3 mm (p < .05)	UIC 6 mm (p < .05)
BVD	0.48	0.58	1.7 $e^{-31}$	4.91 $e^{-15}$
BVC	0.45	1.35 $e^{-52}$	7.14 $e^{-115}$	1.5 $e^{-106}$
BVT	1.1 $e^{-7}$	0.006	1.54 $e^{-41}$	6.76 $e^{-14}$
VPI	1.36 $e^{-22}$	8.26 $e^{-31}$	3.89 $e^{-5}$	.040

**TABLE 2:** SSIM values for 3 mm and 6 mm from both OCT500 and UIC.

SSIM (OCT500)	Complete	AMD	CNV	DR	NORMAL	CSC	RVO	Others
3 mm	0.4835 (0.29–0.60)	0.4513 (0.29–0.55)	0.4754 (0.44–0.52)	0.4923 (0.29–0.59)	0.4834 (0.34–.60)	-	-	-
6 mm	0.4175 (0.16–0.52)	0.4102 (0.30–0.50)	0.4224 (0.38–0.45)	0.4329 (0.35–0.52)	0.4212 (0.25–0.49)	0.4140 (0.32–0.45)	0.3664 (0.26–0.43)	0.4169 (0.16–0.51)

SSIM (UIC)	Complete	Control	Mild	Moderate	Severe
3 mm	0.2808 (0.13–0.39)	0.3188 (0.21–0.38)	0.2961 (0.14–0.35)	0.2914 (0.13–0.39)	0.2647 (0.13–0.39)
6 mm	0.2952 (0.12–0.38)	0.2820 (0.12–0.36)	0.2966 (0.19–0.37)	0.2920 (0.12–0.38)	0.2679 (0.12–0.37)

**TABLE 3:** FID and PCQI scores for the complete datasets of OCT500 and UIC.

OCTA dataset (OCT500)	FID	PCQI (mean, SD)
3 mm	35.88	0.99795 (0.000457)
6 mm	49.06	0.99778 (0.000539)

OCTA Dataset (UIC)	FID	PCQI (Mean, SD)
3 mm	150.34	0.99546 (0.000737)
6 mm	107.74	0.99555 (0.000606)

**TABLE 4:** Statistical analysis of TR-OCTA compared to GT-OCTA for 3 mm dataset from OCT500.

OCTA Dataset	Dataset (no. Of patients)	BVD (mean, SD)	BVC (mean, SD)	BVT (mean, SD)	VPI (mean, SD)
TR-OCTA	Complete (200)	212.31 (29.93)	22.80 (0.81)	1.086 (0.006)	26.91 (5.47)
GT-OCTA		210.22 (29.04)	22.75 (0.41)	1.089 (0.006)	31.43 (2.35)
TR-OCTA	AMD (6)	213.73 (20.05)	22.45 (1.03)	1.087 (0.009)	29.24 (1.91)
GT-OCTA		205.46 (26.45)	22.91 (0.39)	1.09 (0.003)	29.69 (1.63)
TR-OCTA	CNV (5)	228.53 (22.36)	22.34 (1.04)	1.087 (0.003)	26.36 (3.7)
GT-OCTA		224.22 (16.47)	22.90 (0.53)	1.089 (0.005)	30.26 (2.55)
TR-OCTA	DR (29)	209.07 (27.51)	23.12 (0.71)	1.080 (0.007)	26.92 (4.32)
GT-OCTA		210.80 (34.82)	23.14 (0.42)	1.087 (0.005)	28.25 (3.55)
TR-OCTA	NORMAL (160)	212.34 (30.86)	22.77 (0.81)	1.086 (0.006)	26.84 (5.79)
GT-OCTA		209.86 (28.39)	22.67 (0.37)	1.089 (0.006)	32.11 (1.41)

Similarly, for the complete 6 mm dataset we performed T-tests (p < .05) for BVD, BVC, VPI and BVT but only BVD was found to have statistically similar values for both TR-OCTA and GT-OCTA images (Table 1). The 6 mm dataset contained central serous chorioretinopathy (CSC), retinal vein occlusion (RVO) and other retinal pathologies that were absent in the 3 mm dataset (Figures 4E–H). In a comparative analysis (Table 5), BVD for RVO and BVT for AMD patients shows a larger deviation compared to other cases when calculated. Also, BVD and BVT values for translated image follow the trend set by the GT-OCTA. However, BVC, BVT, and VPI were measured having closer values in all cases. When plotted, the distribution of the feature values for TR-OCTA and GT-OCTA, similar to the 3 mm dataset, BVC, VPI and BVD show more outliers compared to BVT and the distribution is similar to the 3 mm dataset. Supplementary Figures S1E–K exhibits feature value distribution for AMD, CNV, CSC, DR, RVO, other retinal pathologies and normal patients and a similar trend of BVD feature having more outliers is observed for diseased as well as normal patients in comparison to other features except RVO.

**TABLE 5:** Statistical analysis of TR-OCTA compared to GT-OCTA for 6 mm dataset from OCT500.

OCTA dataset	Dataset (no. Of patients)	BVD (Mean ± St.d)	BVC (Mean ± St.d)	BVT (Mean ± St.d)	VPI (Mean ± St.d)
TR-OCTA	Complete (300)	210.80 ± 30.45	44.78 ± 1.37	1.087 ± 0.006	24.95 ± 2.97
GT-OCTA		212.34 ± 37	42.91 ± 1.33	1.088 ± 0.007	27.94 ± 3.02
TR-OCTA	AMD (43)	210.13 ± 32.07	44.28 ± 1.28	1.063 ± 0.005	24.56 ± 3.32
GT-OCTA		204.72 ± 35.76	42.93 ± 1.44	1.063 ± 0.007	27.76 ± 3.65
TR-OCTA	CNV (11)	213.11 ± 27.01	45.00 ± 1.03	1.087 ± 0.003	24.06 ± 1.63
GT-OCTA		224.63 ± 46.59	42.59 ± 1.03	1.089 ± 0.007	27.39 ± 3.36
TR-OCTA	CSC (14)	209.66 ± 22.43	45.12 ± 0.96	1.088 ± 0.0064	25.08 ± 1.86
GT-OCTA		215.35 ± 45.51	43.08 ± 0.98	1.088 ± 0.0063	28.59 ± 2.24
TR-OCTA	DR (35)	215.09 ± 28.04	45.12 ± 1.30	1.086 ± 0.0065	26.2 ± 2.95
GT-OCTA		210.66 ± 37.54	43.50 ± 1.18	1.087 ± 0.0068	28.68 ± 3.2
TR-OCTA	RVO (10)	228.13 ± 60.63	44.13 ± 1.15	1.089 ± 0.0079	24.82 ± 1.76
GT-OCTA		239.79 ± 26.76	43.09 ± 0.91	1.087 ± 0.009	27.55 ± 2.89
TR-OCTA	Others (96)	207.14 ± 30.19	44.88 ± 1.34	1.086 ± 0.0062	25.19 ± 3.06
GT-OCTA		213.22 ± 32.52	43.06 ± 1.19	1.088 ± 0.0073	27.74 ± 2.61
TR-OCTA	Normal (91)	211.34 ± 27.64	44.75 ± 1.50	1.087 ± 0.0072	24.5 ± 2.97
GT-OCTA		210.71 ± 39.42	42.53 ± 1.51	1.089 ± 0.0073	27.95 ± 3.13

For UIC dataset Table 2, 3 summarize the qualitative metrics and Tables 6, 7 summarize quantitative feature values for both 3 mm and 6 mm presenting the validity of implementation of automated image-to-image translation. The whole 3 mm dataset show a mean SSIM value of 0.2808 however Control group as well as other DR stages show slight deviation in terms of mean SSIM although their value range stays similar. For the complete 6 mm, SSIM was slightly higher (0.2952) and contrary to the 3 mm set, Control, Mild and Moderate groups show closer values to each other except for Severe with a slight decrease in value: 0.2679. As a quality metrics, FID scores show higher value than OCT500 for both 3 mm and 6 mm: 150.34 and 107.74 respectively however PCQI scores were closer to the ideal value for both 3 mm and 6 mm.

**TABLE 6:** Statistical analysis of TR-OCTA compared to GT-OCTA for 3 mm dataset from UIC.

OCTA Dataset	Dataset (no. Of scans)	BVD (Mean ± St.d)	BVC (Mean ± St.d)	BVT (Mean ± St.d)	VPI (Mean ± St.d)
TR-OCTA	Complete (187)	220.91 ± 21.73	18.21 ± 0.86	1.078 ± 0.0066	24.52 ± 2.70
GT-OCTA		189.29 ± 25.59	20.95 ± 0.65	1.090 ± 0.0086	26.11 ± 4.45
TR-OCTA	Control (35)	212.69 ± 17.66	18.38 ± 0.80	1.083 ± 0.0082	25.48 ± 1.90
GT-OCTA		217.57 ± 27.82	20.25 ± 0.48	1.089 ± 0.0073	31.30 ± 4.53
TR-OCTA	Mild (118)	223.29 ± 22.97	18.00 ± 0.86	1.078 ± 0.0056	23.99 ± 2.76
GT-OCTA		180.42 ± 19.80	21.17 ± 0.58	1.091 ± 0.0088	24.56 ± 3.47
TR-OCTA	Moderate (37)	216.40 ± 24.49	18.51 ± 0.97	1.078 ± 0.0065	24.73 ± 2.45
GT-OCTA		189.60 ± 20.77	20.91 ± 0.47	1.091 ± 0.0102	26.29 ± 3.25
TR-OCTA	Severe (97)	229.30 ± 18.85	17.96 ± 0.95	1.077 ± 0.0061	23.83 ± 2.76
GT-OCTA		181.44 ± 20.79	20.99 ± 0.50	1.090 ± 0.0087	24.51 ± 3.57

**TABLE 7:** Statistical analysis of TR-OCTA compared to GT-OCTA for 6 mm dataset from UIC.

OCTA Dataset	Dataset (no. Of scans)	BVD (Mean ± St.d)	BVC (Mean ± St.d)	BVT (Mean ± St.d)	VPI (Mean ± St.d)
TR-OCTA	Complete (258)	214.75 ± 25.5	37.30 ± 0.93	1.084 ± 0.0059	25.66 ± 2.86
GT-OCTA		199.76 ± 15.05	39.54 ± 0.87	1.089 ± 0.0069	27.14 ± 2.46
TR-OCTA	Control (59)	211.57 ± 21.79	37.35 ± 0.72	1.085 ± 0.0064	28.55 ± 2.19
GT-OCTA		197.61 ± 12.83	39.40 ± 0.74	1.088 ± 0.0062	27.43 ± 2.027
TR-OCTA	Mild (143)	218.19 ± 22.93	37.12 ± 1.067	1.084 ± 0.0056	25.86 ± 2.74
GT-OCTA		199.29 ± 14.16	39.60 ± 0.80	1.088 ± 0.0070	26.76 ± 2.30
TR-OCTA	Moderate (69)	209.54 ± 29.57	37.64 ± 0.83	1.084 ± 0.0060	27.00 ± 2.42
GT-OCTA		204.29 ± 14.62	39.41 ± 0.93	1.089 ± 0.0072	27.81 ± 2.31
TR-OCTA	Severe (123)	216.41 ± 25.25	37.14 ± 0.81	1.083 ± 0.0057	26.12 ± 2.44
GT-OCTA		201.34 ± 14.96	39.34 ± 0.84	1.089 ± 0.0072	26.72 ± 2.52

**FIGURE 6:**
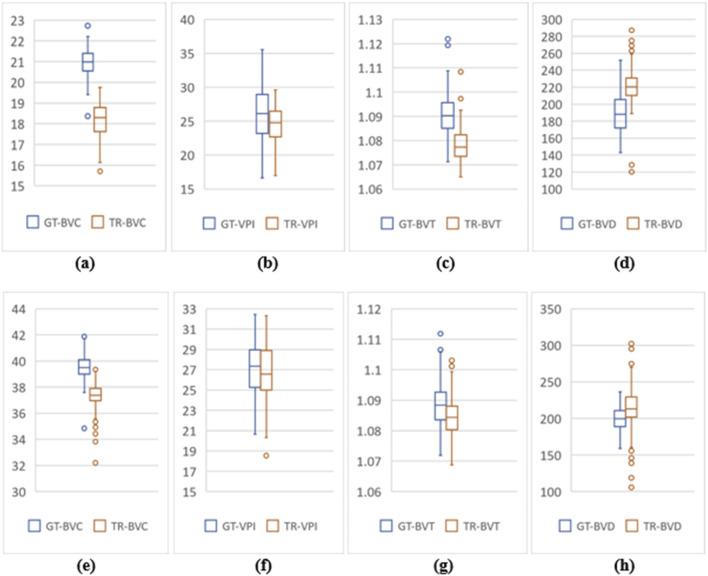
Feature value distribution of 3mm and 6 mm scans for the UIC dataset. **(A–D)** are BVC, VPI, BVT, and BVD values for 3 mm. Similarly, **(E–H)** show values for 6 mm scans.

From two-tail T-tests (p < .05) BVD, BVC, BVT and VPI values for TR-OCTA were found to be statistically different from GT-OCTA for 3 mm and 6 mm which is expected due to the fact that UIC data was excluded from the training (Table 1). These feature values were calculated (3 mm and 6 mm) for the complete dataset as well as separately for different patient groups. For 3 mm (Table 6), BVD (Figures 4I–L) shows more deviation from the GT values compared to BVC and VPI except BVT which shows better comparative values. From Figure 6 we can observe the difference between TR and GT images and concentrated values with some outliers for BVC, BVT, and BVD. Supplementary Figures S2A–D shows the range of values for all the features observed in different DR groups and the mean values for TR-OCTA lies below the GT-OCTA most of the cases except for BVD which lies above GT values for Mild, Moderate and Severe group.

A similar pattern is recognized from the 6 mm analysis (Table 7) having BVD a higher deviation from the GT compared to BVC, BVT, and VPI. BVD shows more outliers for TR-OCTA than other features contributing to the larger difference (Figure 6). All these feature values are calculated separately for Control, Mild, Moderate and Severe groups and in general BVD shows higher difference between TR-OCTA and GT-OCTA among all the features for different patient groups (Figures 4M–P). Supplementary Figures S2E–H represents feature value distribution with outliers for different DR groups. Similar to 3 mm, TR-OCTA BVD feature value distribution shows a mean higher than the GT-OCTA for Control, Mild, Moderate and Severe groups.

## Discussion

In this paper, we implemented a recently demonstrated algorithm [36] for OCT-OCTA translation and validated the translated OCTA images to show their utility in quantitatively characterizing retinal features using two datasets OCT500 and UIC. We present a comprehensive analysis comparing the performance of GT- OCTA images with those generated by a TR-OCTA across different patient groups, including those with complete data sets, for both 3 mm and 6 mm FoV. Several qualitative (SSIM, FID, PCQI) and quantitative metrics (BVD, BVC, BVT, and VPI) were considered to validate the comparative performance analysis on a clinical dataset from UIC. We found FID and PCQI scores to be the most reliable qualitative metrics and a combination of BVT, VPI could be considered best for distinguishing diseases specially DR patients in TR-OCTA.

SSIM was utilized as a quality metric to assess the similarity between TR-OCTA and GT-OCTA images, providing insight into the translation model’s ability to replicate key structural features of the retinal vasculature. For OCT500, between 3 mm and 6 mm, mean SSIM from the complete dataset of 3 mm was higher than 6 mm despite both values were far from the ideal value. The reason behind is how the translation algorithm works that focuses on the vasculature rather than the entire image while SSIM considers pixel difference between the reference and the target image for its entirety. This becomes more prominent from the UIC dataset. Both 3 mm and 6 mm sets form UIC shows lower mean SSIM than OCT500 since the algorithm was exclusively trained on OCT500 and as a result the generated TR-OCTAs show less structural similarity overall to the GT-OCTA. SSIM for different patient groups was also considered to measure the response of the TR-OCTAs for different pathologies. UIC dataset mostly show consistent mean SSIM across different pathologies except for RVO (6 mm) indicating the algorithm couldn’t capture the vascular structure from the RVO patient equally as other pathologies which could also be contributed to the lower number of sample available for training (3.34%). Similar observations can be made from UIC 3 mm and 6 mm however the model fails to capture the key structural features of severe stage DR patients. Therefore, from our observations, SSIM is not an ideal metric for GAN generated OCTA images.

Two more quality metrics, FID and PCQI scores were considered as these are more suitable for GAN generated image quality comparison against reference (GT) images. OCT500 3 mm set, having lower FID score, indicate higher similarity to the reference data, suggesting that the 3 mm scans exhibit better image quality compared to the 6 mm scans within this dataset. This trend is consistent with expectations, as higher resolution (smaller scanning area) typically results in finer detail and less distortion. The UIC dataset shows comparatively higher FID scores for both 3 mm and 6 mm scans, indicating lower fidelity compared to the OCT500 dataset which can be explained as the effect of implementing the model to a dataset excluded from the training. Interestingly, the 6 mm scans have a lower FID score than the 3 mm scans, suggesting better relative performance at a larger scanning area for this dataset. This inverse trend can be attributed to variations in image quality for 6 mm image acquisition (image collected after a certain date is of higher quality than the previous scans). Another quality metric, PCQI values for the OCT500 dataset are found to be exceptionally high for both 3 mm and 6 mm. These results indicate minimal variation and high consistency in image quality across different scans. The slight decrease in PCQI for the 6 mm scans is consistent with the increased FID score, further confirming that the 3 mm scans offer superior quality. The PCQI scores for the UIC dataset, while slightly lower than those of the OCT500 dataset, still demonstrate high image quality, for 3 mm and 6 mm sets. The minimal difference in PCQI between 3 mm and 6 mm scans suggests that the image quality is relatively stable across different FoVs, despite the higher FID scores.

To compare the quantitative feature values between TR-OCTA and GT-OCTA, we focus on the performance across different categories since neither OCT500 nor UIC has equal distribution of data across different pathologies and normal or control group. In a comparative analysis from OCT500, BVD shows more deviation from the GT-OCTA, specially for 6 mm, supporting the better FID and PCQI scores for 3 mm, indicating the model can capture the superficial layer vasculature better than the deep layer hence vessel density shows bigger difference between TR-OCTA and GT-OCTA (Tables 4, 5). This feature also has the greatest number of outliers (Figure 5) among all features contributing to larger difference. Similar observations can be made from UIC dataset, showing the models limitation in capturing vasculature from all layers equally. When plotted, the trend set by the GT-OCTA for BVD show higher deviation for CNV in OCT500 3 mm. TR-OCTA also follows the same trend, however, for OCT500 6mm, BVD feature values tend to follow the GT-OCTA apart from RVO patients. On the other hand, for UIC 3 mm and 6 mm, BVD fails to properly exhibit the trend shown by GT-OCTA indicating inclusion of the data in the training process provides better result for BVD (Tables 6, 7). However, for UIC 6mm, BVD shows clear distinction between control and early stage DR as well as severe stage DR, similar to what has been reported in other studies [12]. Overall, when trained, BVD could be considered as a potential biomarker for CNV and RVO patients as evident from the analysis (Figure 4).

BVC, another quantitative feature, unlike BVD, doesn’t show that large of a deviation from the GT values across different pathologies indicating it could be used as a measure of performance even on a dataset not seen before. OCT500 3 mm TR-OCTA tend to show some variations for CNV and RVO patient similar to BVD however the trend goes beyond what is observed from GT-OCTA. From UIC dataset, however, BVC feature values across different pathologies stay in close range across different stages of DR patients without showing any large deviation. However, the TR-OCTA values follow GT-OCTA trend.

BVT feature values show least deviation for all datasets across different patient categories. For OCT500 3 mm, TR-OCTA values follow the trend set by GT-OCTA specially showing a clear distinction among DR and other pathologies as well as normal state (Figure 4C). In consistent with this scenario, UIC BVT feature values stay in close range for (TR-OCTA and GT-OCTA) 3 mm and show a better trend line for 6 mm. From OCT500 6 mm, BVT feature values, while closely following GT-OCTA values, show clear distinction for AMD patients indicating a potential choice for AMD classification at lower FoV.

Finally, VPI feature values for OCT500 3 mm TR-OCTA show an opposite trend compared to GT-OCTA. GT-OCTA show clear distinction among AMD, CNV and DR pathologies however TR-OCTA fails to identify the distinction. However, OCT500 6 mm TR-OCTA shows a much better performance showing the clear deviation of values for CNV, CSC and DR patients indicating the model’s ability to distinguish between normal and AMD, CNV, DR patients in general. A similar picture is depicted by the performance analysis on UIC dataset, specially 3 mm, clearly identifying different DR stages from control groups indicating VPI as a better choice of potential biomarker for DR patients.

Overall, in light of the comparative analysis performed on both datasets, SSIM values were higher for OCT500 rather than UIC indicating the inclusion of the prior dataset affecting the quality of the generated images. Similar scenarios can be observed from FID scores, OCT500 having lower value hence better resemblance to the GT-OCTA images. However, PCQI scores for both datasets (OCT500 and UIC) indicates the TR-OCTA images are almost the same as the GT-OCTAs in terms of contrast and sharpness which is supported by our analysis that some features (BVT, VPI) show better performance across both FoV. Although, feature values for BVC and VPI showed slight variation, they were still in close proximity for both OCT500 and UIC. BVT, however, is found having almost consistent values for both dataset in all cases. BVD, on the other hand, shows bigger variation between TR-OCTA and GT-OCTA for UIC compared to OCT500, an expected outcome similar to SSIM, as density features tend to be affected by local vasculature more than the vessel features.

Despite a better performance from the TR-OCTA across the datasets, our study has some inherent limitations. One limitation being the scarcity of the publicly available data, restricting the ability to perform extensive and varied analyses. Additionally, the dataset used in this research is relatively small, particularly when considering cross-pathological studies. This limited sample size can hinder the generalizability of the findings. Furthermore, our data distribution is unequal across different patient categories and pathologies, potentially introducing bias and affecting the robustness of our conclusions. Another significant challenge is the inconsistency in image quality and signal strength. Not all OCTA images have the same signal strength or quality, which can adversely impact the performance of translated OCTA in both quantitative and qualitative assessments. These limitations highlight the need for more comprehensive datasets and improved image acquisition standards to enhance the reliability and applicability of OCTA feature studies. Although similar studies have been conducted [12, 14] on OCTA features, to our knowledge, this is the first study conducted on quantitative characterization and extensive comparative analysis of retinal features for TR-OCTA images. We tried to establish the idea that while the translation model holds promise in reproducing retinal vasculature across various conditions, there exist minor variations in the accuracy of vascular metrics between TR-OCTA and GT-OCTA images. These discrepancies underscore the necessity for ongoing enhancements to the translation model to achieve higher precision in vascular representation, particularly for pathological conditions where accurate vascular depiction is critical for clinical diagnosis and monitoring.

This study showcases the potential of AI to bridge the gap between OCT’s inability to visualize blood flow information and leveraging generative-adversarial learning frameworks for image translation to capture that information. Our findings suggest that AI-driven translation models can generate high-quality OCTA images from OCT data (demonstrated using SSIM, FID and PCQI metrics) and the quantitative features generated in TR-OCTA follow a similar trend as in GT-OCTA. This has the potential to significantly improve the accuracy and efficiency of diagnosing and monitoring retinal diseases through OCTA imaging, emphasizing the need for further research and development in this area.

In summary, this study demonstrates the potential of generative AI in enhancing OCT imaging for ophthalmic diagnostics. By validating quantitative features to check the viability of TR-OCTA, this research addresses significant limitations in widespread adoption of OCTA in clinical settings. Despite facing challenges such as generalization for different retinal diseases and difficulty in capturing detailed vascular networks, our study lays a solid foundation for future advancements in multi-modal OCT based retinal disease diagnosis and monitoring. The incorporation of AI not only promises to reduce the dependence on costly OCTA devices but also opens new avenues for accessible and accurate retinal healthcare solutions. Moving forward, it is imperative to refine these AI models to improve the resolution and accuracy of translated OCTA images, ensuring they can reliably support clinical decision-making and contribute to the broader understanding of retinal pathologies.

## Data Availability

Publicly available datasets were analyzed in this study. The OCT500 dataset can be found here: https://ieee-dataport.org/open-access/octa-500. UIC data can be requested to the corresponding author.

## Supplementary material

SUPPLEMENTARY FIGURE S1
**(A–D)** show BVC, VPI, BVT, and BVD feature values for the 3 mm dataset (OCT500) with different patient conditions. a(i-iv) are AMD patients, b(i-iv) are CNV patients, c(i-iv) are DR patients. d(i-iv) are Normal patients. **(E–K)** show feature values for the 6 mm dataset (OCT500) with different patient conditions. e(i-iv) are AMD patients, f(i-iv) are CNV patients, g(i-iv) are CSC patients, h(i-iv) are DR patients, i(i-iv) are RVO patients, j(i-iv) are patients with other retinal pathologies, k(i-iv) are Normal patients.

SUPPLEMENTARY FIGURE S2
**(A–D)** show BVC, VPI, BVT and BVD for the UIC 3 mm scans with different patient conditions. a(i-iv) shows Control group, b(i-iv) Mild, c(i-iv) Moderate, d(i-iv) Severe patient scans. Similarly, **(E–H)** show BVC, VPI, BVT and BVD for the UIC 6 mm scans with different patient conditions. e(i-iv) for Control group, f(i-iv) for Mild, g(i-iv) for Moderate, h(i-iv) for Severe patient scans.
