# Supplementary figures and images for: Quantitative characterization of retinal features in translated OCTA
Source: Exp Biol Med (Maywood). 2024 Oct 23;249:10333. doi: 10.3389/ebm.2024.10333 (PMC11537946; doi:10.3389/ebm.2024.10333)

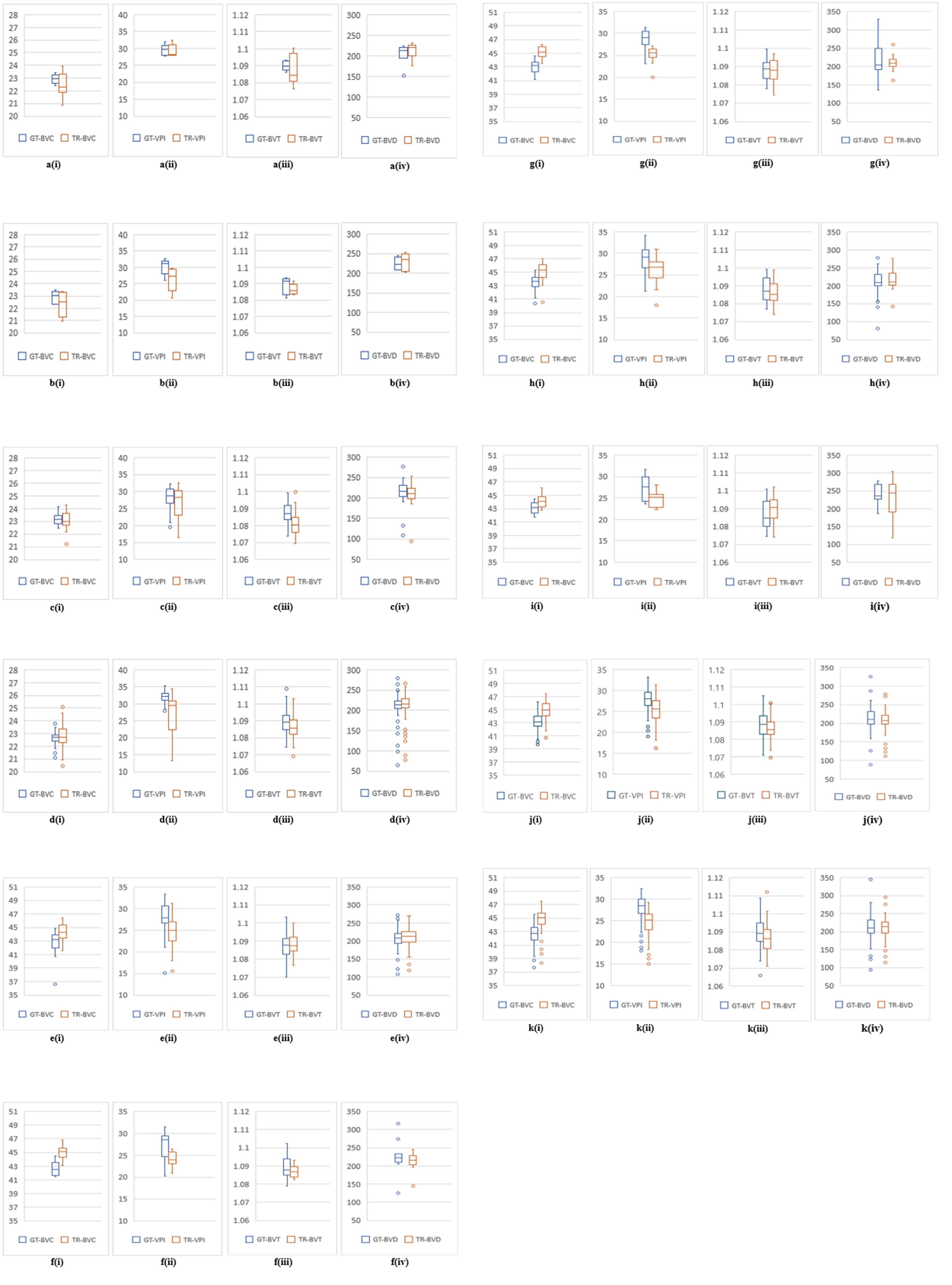

Supplement: Supplementary file 1 [file Image1.JPEG]

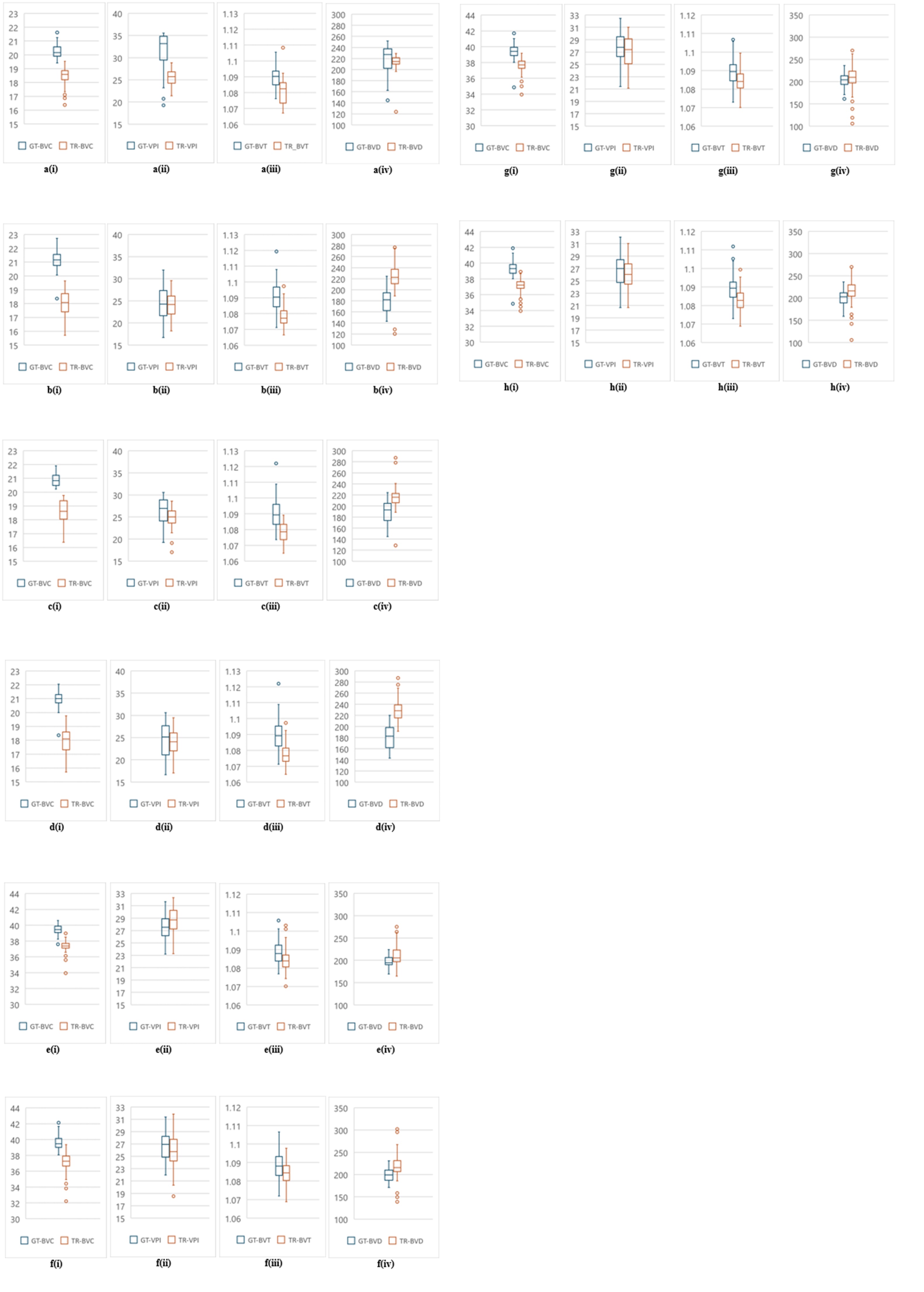

Supplement: Supplementary file 2 [file Image2.JPEG]
